# Safety of CAR-T Cell Therapy in Patients With Renal Failure/Acute Kidney Injury: Focused Review

**DOI:** 10.1007/s44228-023-00037-7

**Published:** 2023-04-03

**Authors:** Israr Khan, Nida Khan, Natalie Wolfson, Kawthar Djebabria, Mohammad Ebad Ur Rehman, Faiz Anwer

**Affiliations:** 1 Department of Internal Medicine, HMH Palisades Medical Center, 7600 River Rd, North Bergen, NJ 07047 USA; 2 Department of Internal Medicine, Jinnah Sindh Medical University, Karachi, Pakistan; 3 Department of Internal Medicine, Touro College of Osteopathic Medicine Harlem, OMS-III, New York, NY USA; 4 Department of Internal Medicine, Annaba’s University Hospital, Annaba, Algeria; 5 Department of Internal Medicine, Rawalpindi Medical University, Rawalpindi, Pakistan; 6 Department of Hematology and Medical Oncology, Taussig Cancer Institute, Cleveland Clinic, Cleveland, OH USA

**Keywords:** Chimeric antigen receptor T-cell therapy, CART, Adoptive immunotherapy, Acute kidney injury, Renal insufficiency, Renal impairment, Diffuse large B-cell lymphoma

## Abstract

Chimeric antigen receptor (CAR) T-cell therapy is novel immunotherapy targeting specifically cancerous cells, and has been shown to induce durable remissions in some refractory hematological malignancies. However, CAR T-cell therapy has adverse effects, such as cytokine release syndrome (CRS), immune effector-associated neurotoxicity syndrome (ICANS), tumor lysis syndrome (TLS), and acute kidney injury (AKI), among others. Not many studies have covered the repercussions of CAR T-cell therapy on the kidneys. In this review, we summarized the available evidence on the safety profile of CAR T-cell therapy in patients with pre-existing renal insufficiency/AKI and in those who develop AKI as a result of CAR T-cell therapy. With a 30% incidence of AKI post-CAR T-cell, various pathophysiological mechanisms, such as CRS, hemophagocytic lymphohistiocytosis (HLH), TLS, serum cytokines, and inflammatory biomarkers, have been shown to play a role. However, CRS is commonly reported as an underlying mechanism. Overall, 18% of patients in our included studies developed AKI after receiving CAR T-cell therapy, and most cases were reversible with appropriate therapy. While phase-1 clinical trials exclude patients with significant renal toxicity, two studies (Mamlouk et al. and Hunter et al.) reported successful treatment of dialysis-dependent patients with refractory diffuse large B-cell lymphoma, and demonstrated that CAR T-cell therapy and lymphodepletion (Flu/Cy) can be safely administered.

## Introduction

Chimeric antigen receptor (CAR) T-cell therapy is novel immunotherapy, targeting specifically cancerous cells, and inducing durable remissions in some refractory hematological malignancies like multiple myeloma (MM) [1]. This therapy is promising, as demonstrated in several clinical trials [2]. With its relative safety and efficacy, the Food and Drug Administration (FDA) approved the clinical use of different CAR T-cells, including idecabtagene vicleucel in MM [3], axicabtagene ciloleucel in diffuse large B-cell lymphoma [4], ciltacabtagene autoleucel [5], brexucabtagene autoleucel, lisocabtagene marleucel, and tisgenlecleucel.

CAR T-cell therapy has side effects which include tumor lysis syndrome (TLS) [6], immune effector-associated neurotoxicity syndrome (ICANS) [7], hemophagocytic lymphohistiocytosis (HLH)/ macrophage activation syndrome (MAS) [8], and cytokine release syndrome (CRS) [9], which remarkably affects kidneys. CRS caused by T-cell activation and subsequent release of large amounts of cytokines is experienced by the majority of patients in clinical trials with CAR T-cell therapy [10]. Hypotension, nausea, fever, hypoxia, tachypnea, tachycardia, and pulmonary edema that leads to intravascular depletion are the main clinical presentations of CRS. These cause hemodynamic changes leading to reduced renal flow, ischemia, hypovolemia, and triggering pre-renal AKI [11]. In a case series conducted by Gupta et al., a total of 78 adults received CAR T-cell therapy, and 15 of 78 (19%) developed acute kidney injury (AKI), among which 8 (53%) were due to decreased renal perfusion (resolved in 72 h), 6 (40%) were consequent to acute tubular necrosis (ATN), and 1 developed urinary obstruction. The 60-day mortality and length of hospital stay were higher in those with ATN and obstructive AKI [12].

Similarly, in a retrospective review by Gutgarts et al., 46 adult patients with non-Hodgkin lymphoma (NHL) received treatment with CAR T-cell therapy and reported a cumulative incidence of any grade AKI of 30%, with grades 1/2 and 3 AKI incidences of 21.7%, and 8.7%, respectively [13]. Moreover, no patients developed severe AKI necessitating renal replacement therapy, alongside satisfying kidney function recovery by day 30. That said, phase-1 trials exclude patients with significant renal toxicity. Herein, we aim to summarize all available evidence on the safety profile of CAR T-cell therapy in patients with renal insufficiency/AKI, and to provide insights to encourage the possibility of enrolling this subset of the population in randomized controlled trials and cohort studies.

## Methods

We performed a literature review using PubMed, Cochrane databases, and Google Scholar using the following keywords: CAR T-cell therapy, adoptive immunotherapy, renal failure, end-stage renal disease, and acute kidney injury. Articles suitable for extraction from the literature searches were case report, case series, editorials, and retrospective reviews. We included studies published in English and reporting data on the safety of CAR-T cell therapy in patients with AKI or renal failure. Non-human studies and studies in languages other than English were excluded. There are currently no published randomized controlled trials available in the defined population. We reviewed the relevant findings and added the information to the data extraction sheet. We used pooled analysis to report some commonly reported data across studies.

## Results

### Baseline Characteristics

A total of 252 patients (male = 166, female = 86) included in the analysis were treated with CAR T-cell therapy and enrolled across 9 studies from 2016 to 2021. The mean age of the sample was 48 (19–86). The diseases treated with CAR T-cell therapy included NHL (*n* = 129), diffuse large B-cell lymphoma (DLBCL, *n* = 120), post-transplant lymphoproliferative disorder (PTLD, *n* = 1), B-cell ALL (*n* = 1) and Burkitt Lymphoma (*n* = 1). Cyclophosphamide (Cy) with fludarabine (Flu) is a common non-myeloablative conditioning regimen and was utilized in 6 of the studies. One study added rituximab to Cy/Flu for one patient, and another reported only using bendamustine for three patients. Three of the studies did not report their conditioning regimen. The type of CAR T-cell therapy used in the analysis includes tisagenlecleucel alone (Lee et al., Melilli et al., Acharya et al., De Nattes et al.), tisagenlecleucel and axicabtagene ciloleucel (Gupta et al., Gutgarts et al.), axicabtagene ciloleucel alone (Farooqui et al. and Mamlouk et al.), axicabtagene ciloleucel and lisocabtagene maraleucel (Hunter et al.). In some cases, due to persistent CD19 antigen, the patient received a 28-day course of a bispecific CD19-directed CD3 T-cell engager antibody construct (blinatumomab), started 6 weeks after the third CAR-T cell infusion, and stopped after negative CD19 antigen at the time of course completion (Acharya et al.).

Baseline creatinine before CAR T-cell therapy ranged from 0.5 to 2 mg/dL. The mean creatinine values pre-therapy for all nine studies was 0.8 mg/dL. In one study, two subjects had ESRD and were receiving hemodialysis (Hunter et al.) AKI was described in 70% of the studies, with the exception of Hunter et al.’s, where creatinine level was not reported due to concomitant ESRD. Table 1 shows patients’ characteristics and kidney profiles before and after receiving CAR-T cell therapy, respectively.

**Table 1:** Patient’s characteristics and kidney profile before and after administering chimeric antigen receptor T-cells (CAR-T cells)

Author	Patients (N)	Mean Age (range)	Condition	Type of CAR T- cells	Kidney function before CART	Kidney function after CART	AKI
Gutgarts et al. [13]	*N* = 46	63 (19–68)	Non-Hodgkin lymphoma	Axicabtagene ciloleucel & Tisagenlecleucel	Cr: 0.8 (0.5–2) mg/dL, GFR 88 [ 36-160 mL/min/1.73m2	Cr: 1.1 mg/dl ( 0.9–2.1 mg/dl)	AKI:14/46 (30%)
Lee et al. [49]	*N* = 37	60	Diffuse large B-cell lymphoma	Tisagenlecleucel	Cr: 0.54 mg/dL, BUN: 19 mg/dL	Cr: 1.36 mg/dL, BUN: 44 mg/dL	AKI:2/37 (5%)
Gupta et al. [12]	*N* = 78	60 ± 13	Diffuse large B-cell lymphoma	Axicabtagene ciloleucel & tisagenlecleucel	Cr: 0.8 mg/dL	Cr: 1.4 mg/dL	AKI:15/78 (19%)
Hunter et al. [14]	*N* = 2	52–66	Relapsed/refractory large B-cell lymphoma	Axicabtagene ciloleucel, Lisocabtagene maraleucel	NA	NA	AKI: N/A
Farooqui et al. [60]	*N* = 83	55.2	Non-Hodgkin lymphoma	Axicabtagene ciloleucel	Cr:0.8 mg/dL, eGFR: 91.3 mL/min/ 1.73m2	NA	AKI:14/83 (17%)
Melilli et al. [47]	*N* = 1	40	Monomorphic typeB-cell posttransplant lymphoproliferative disorder	Tisagenlecleucel	Cr: 1.5 mg/dL	Cr: 6 mg/dL	AKI:1/1 (100%)
Mamlouk et al. [15]	*N* = 3	38–44	Diffuse large B-cell lymphoma	Axicabtagene ciloleucel	Cr: 1.20, 0.74, and 1.53 mg/dL	Cr: 1.5, 0.7, 1.7 mg/dL	NA
Acharya et al. [48]	*N* = 1	20	Relapsed/refractory Pre-B-cell ALL	Tisagenlecleucel	Cr: 0.52 mg/dL	Cr:2.91 mg/dL, GFR:27 mL/min/1.73 m2	AKI:1/1 (100%)
Nattes et al. [17]	*N* = 1	40	Refractory Burkitt lymphoma	Tisagenlecleucel)	Cr 0.8 mg/dL	Cr 11.1 mg/dL decrease to 1.5 mg/dL after steroids	AKI:1/1 (100%)

*AKI* Acute kidney injury, *NA* Not available, *Cr* creatinine, *eGFR* Estimated glomerular filtration rate, *N* number of patients

### Clinical Outcomes

The average creatinine levels after therapy ranged between 0.8 and 2 mg/dL, with one patient presenting with 6 mg/dL. For another patient who presented with levels of 11 mg/dL, steroids helped decrease this to 1.5 mg/dL. The CAR T-types which are effective in renal disease are axicabtagene ciloleucel and lisocabtagene maraleucel. It was usually preceded by lymphodepletion chemotherapy with Flu and Cy. Among 252 patients, 46/252 (18%) developed AKI after receiving CAR T-cell therapy. However, 80% developed CRS following CAR T-cell therapy in the included studies, while *n* = 135/250 (54%) developed ICANs. Most cases of AKI were reported with the use of axicabtagene ciloleucel therapy. We also found that AKI resolved in most cases with supportive care and adequate hydration.

## Discussion

CAR T-cell therapy was highly effective in patients with renal failure, both with and without pre-existing kidney disease. It can be safely administered with lymphodepletion chemotherapy. According to Hunter et al. [14], patients with end-stage renal disease achieved complete response post CAR T-cell therapy, making it a strong consideration in treating renal disease patients. In our pooled analysis, about 18% developed AKI post-CAR T-cell therapy, whereas most patients reported improved blood urea nitrogen and creatinine levels post-therapy. Three patients from the Mamlouk et al. [15] study, received prior renal transplant for other causes, and thus were excluded from the analysis. Additionally, baseline renal function was not reported in Hunter et al. [14] due to both patients having been diagnosed with ESRD before starting. The improved renal function and kidney injury in patients with pre-existing renal failure emphasize the safety of CAR T-Cell therapy in renal failure.

According to one study [18], there is low renal toxicity for anti-B-cell maturation antigen (BCMA) CAR T-cell therapy. Another clinical trial illustrated safe renal outcomes for MM patients post-CAR T-cell therapy [19]. CD8 + CAR T-cells can target the autoreactive pathogenic B cells. The CAAR T-cells (chimeric autoantigen receptor T cells) that express relevant autoantigens can attract autoreactive B cells and target them successfully. These experiments provide a wide array of the possible efficacy of CAR T-cell therapy in kidney transplantation and immune diseases [20].

Post CAR T-cell therapy, various pathophysiological mechanisms have been shown to play a role, such as CRS, HLH, tumor lysis syndrome, serum cytokines, and inflammatory biomarkers [12, 13, 16]. CRS is usually considered the main cause of kidney injury post-therapy due to the underlying cytokine cascade, which increases third spacing, worsening hypotension, and vascular permeability, while the circulating cytokines cause cardiac toxicity leading to diminished cardiac output and cardiorenal syndrome. Acute cardiomyopathy from CRS promotes hypotension and exacerbates hypoperfusion in the kidney [8, 9, 11]. Hypotension, nausea, fever, hypoxia, tachypnea, tachycardia, and pulmonary edema that leads to intravascular depletion are the main clinical presentations of CRS. CRS can cause hemodynamic changes leading to reduced renal flow, ischemia, hypovolemia, and triggers pre-renal AKI [11, 16]. The prolonged decreased kidney perfusion and pre-renal AKI then progresses to acute tubular necrosis (ATN) [12, 21]. Of note, in Nattes et al.’s case report, the etiology of kidney damage, AKI, and transplant rejection might have been due to either CAR T-cell therapy or T-cell-mediated rejection [17]. According to Teachey et al., specific cytokines, including IL-6, soluble IL-6 receptor, interferon-γ, and soluble gp130, are major predictors of CRS after CAR T-cell therapy [22]. Cytokines may directly affect the kidney via intra-renal inflammation and have direct tubular toxicity [23]. IL-6 has been implicated as a key factor in developing systemic adverse effects in CRS [23]. In the case of AKI and chronic kidney disease (CKD), IL-6 increases fibroblast growth factor 23 levels, which may contribute to phosphaturia and hypophosphatemia, thus affecting renal function [24].

Patients receiving CAR T-cell therapy can develop fulminant HLH and present with increased lactate dehydrogenase, hyperuricemia, IL-10, IL-6, and IFN-γ. The ATN, acute interstitial nephritis (AIN), and thrombotic microangiopathy related to HLH, together with capillary leakage and cytokine-mediated vasodilation, trigger pre-renal ischemia [25, 26]. TLS after CAR T-cell therapy for refractory chronic lymphocytic leukemia is also a cause of AKI, according to Porter et al. [27]. In TLS, the damage to large amounts of tumor cells leads to rapid release of intracellular substances such as potassium, uric acid, calcium, and phosphorus results in a series of metabolic disorders [28]. The treatment with anti-CD19-CAR T cells leading to TLS causes phosphate and uric acid to precipitate and block renal tubules leading to renal tubular injury [29]. Another proposed mechanism is when cytokines produced by infiltrated interstitial and glomerular lymphocytes after CAR T-cell therapy activate podocytes and renal tubular epithelial cells [30]. In turn, podocytes increase cytokine production, such as tumor necrosis factor α, IL-8, and IL-6, which leads to kidney injury [31].

While there have been isolated cases of severe renal impairment post Flu exposure [37–39], most are in context of TLS, and the incidence of AKI remains less than 5% [36]. Special considerations for patients with pre-existing renal impairment given Flu treatment should be addressed, as 60% of each Flu dose is excreted in the urine [35]. Dose-reduced Flu (Table 2) has shown reasonably equivalent exposure while maintaining a higher index of safety [35]. Despite occasions of creatinine increasing up to threefold, the worsening renal function is amenable to recovery with proper hydration [32]. One retrospective study has shown that dose-reduced Flu in cases of underlying renal impairment does not affect PFS and OS when compared to patients with previously normal renal function [33]. Thus, lymphodepletion dosing regimens can be personalized to avoid renal failure without compromising eligibility or response to CAR-T cell therapy. However, it is important to note that a new study has shown optimal lymphodepletion dosing (greater than 13.8 mg x h/L) results in lower relapse rates and improved survival after CAR-T cell therapy [34]. There is no clear consensus on exactly how much to reduce Flu doses in patients with underlying renal impairment. Most studies suggest a 20–25% reduction for mild impairment and up to a 50% for moderate to severe impairment [40–42].

**Table 2:** Fludarabine dose recommendation in patients with renal insufficiency

Author/references	Dose reduction
Bodge et al. [40]	20–25% reduction for GFR between 45–89 ml/min/1.73m^2^ 50% reduction for GFR between 44–29 ml/min/1.73m^2^ and those on HD *based on KDIGO guidelines
Golightly et al. [41]	20% reduction CrCl 50–79 mL/min 40% reduction CrCl 30–49 mL/min Do not administer for CrCl < 30 mL/min
Aronoff et al. [42]	25% reduction for CrCl 10–50 mL/min 50% reduction for CrCl < 10 mL/min

*CrCl* Creatinine clearance, *GFR* Glomerular filtration rate, *HD* Hemodialysis *KDIGO Kidney Disease Improving Global Outcome

Renal damage after CAR-T cell therapy is often witnessed. In all the trials of CD-19–directed CAR-T cells, more than 40% of patients depicted CRS regardless of the CAR-T cell construct or the disease [27, 43–45]. The rise in serum creatinine was evident, particularly 7–10 days post-infusion, [27, 45, 46]. This was similar in patients with Burkitt lymphoma and TLS has also been evident in the initial trials of CAR-T cell therapy for chronic lymphocytic leukemia, where elevations in uric acid, lactate dehydrogenase, phosphorus levels, and AKI were noted around 22 days after CAR-T cell infusion [27].

Despite the post-CAR T-cell-induced renal damage, patients can present with borderline AKI before receiving the therapy, and their creatinine stays within manageable range, especially with axicabtagene ciloleucel [15, 47]. The majority of patients with B-cell lymphomas also present with pre-CAR-T cell therapy AKI [13], though the incidence of AKI after CAR-T cell therapy is low, unless an ICU admission or grade 3–4 CRS occurs [13].

Kidney complications are associated with various cancer therapies, especially CAR T-cell therapy. Each CART product must be evaluated individually for its toxicity profile to minimize adverse events [12, 49]. Axicabtagene ciloleucel is associated with higher toxicity, and indirectly increases the risk of AKI through CRS mechanisms [50]. Tisagenlecleucel has a reduced inflammatory profile, lower toxicity rate, and low rates of AKI (5%) [49, 51]. The two series evaluating patients who received axicabtagene ciloleucel found higher rates of AKI (23%), severe CRS (13%), and overall CRS (83%) [12, 13].

Previous nephrotoxic medication exposures before post-CART AKI development are common in these patients and include medications like vancomycin, acyclovir, ibuprofen, trimethoprim-sulfamethoxazole, and intravenous contrast. Patients developing AKI usually receive tocilizumab for CRS and dexamethasone [13]. The pre-renal AKI cohort receives supportive care, avoids nephrotoxic agents, uses intravenous fluids and hemodynamic support, with the condition resolving within 72 h [12]. In comparison, ATN and obstructive AKI have high mortality rates [12]. Kidney replacement therapy is mostly fatal, especially in ATN, but post-renal AKI is responsive to IV fluids [12, 13].

While mild and moderate CRS are self-limiting and can be managed with supportive care [52], severe CRS requires corticosteroids, tocilizumab, or siltuximab, either with corticosteroids or alone [52, 53]. Tocilizumab is also indicated for catecholamine-dependent vasodilatory shock and severe CRS. It improves blood pressure and prevents multiple organ failure [54]. Gutgarts et al. [13] reported normal kidney function within 30 days post-CAR T-cell therapy in patients with NHL. Similarly, Hunter et al. [14] reported the successful treatment of two dialysis-dependent patients diagnosed with refractory-DLBCL with CAR T-cell therapy.

To prevent pre-renal AKI in these patients, intravenous fluids, and vasopressors to maintain renal perfusion and systemic hemodynamics are helpful [55]. In TLS, hydration, allopurinol, and alkalinization prevent AKI for low-risk patients, while in the high-risk cohort, hydration and rasburicase help lower uric acid levels, [56]. HLH cases require aggressive immunosuppression involving corticosteroids and anti-IL-6 therapy (tocilizumab or siltuximab) with supportive care [57]. If immunosuppression fails to reduce toxicity, etoposide should be considered [57, 58]. For HLH management, intravenous immunoglobulin has also been recommended [59]. There is a need to identify various biomarkers that can not only predict AKI earlier but can assist in categorizing long-term risks for CKD in patients with post-CAR T-cell therapy [12, 49].

## Conclusion

The incidence of AKI in those receiving CAR T-cell therapy ranges from 50 to 90%. The range is determined by the types of CAR T-cells used and the underlying pathophysiological mechanisms. These include CRS, HLH, TLS, and inflammatory cytokines. Also, other factors, such as lymphodepleting therapy (fludarabine), dehydration, and nephrotoxic agents, may potentiate pre-renal or other renal injuries. Though phase 1 clinical trials exclude patients with significant renal toxicity, two studies have demonstrated that CAR T-cell therapy and lymphodepletion (Flu/Cy) can be safely administered. While there is no clear consensus on how much to reduce Flu doses in patients with underlying renal impairment, most studies suggest a 20–25% reduction for mild impairment and up to a 50% reduction for moderate to severe impairment, without significantly compromising the response to CAR T-cell. Additionally, the majority of post-CAR T-cell therapy-related AKI remains reversible by treating the underlying cause, adequate hydration, supportive care, and tocilizumab or siltuximab. That being said, the safety data we present in the defined population are limited; however, they should pave the way for including patients with renal insufficiency in prospective cohort studies and clinical trials of CAR T-cell therapy to determine its long-term safety and efficacy profiles.

## Abbreviations

CAR T-cell therapy: Chimeric antigen receptor T-cell therapy
CRS: Cytokine release syndrome
ICANS: Immune effector-associated neurotoxicity syndrome
TLS: Tumor lysis syndrome
AKI: Acute kidney injury
ATN: Acute tubular necrosis
MM: Multiple myeloma
NHL: Non-Hodgkin lymphoma
DLBCL: Diffuse large B-cell lymphoma
PTLD: Post-transplant lymphoproliferative disorder
ALL: B-cell acute lymphoblastic leukemia
Cy: Cyclophosphamide
Flu: Fludarabine
CKD: Chronic kidney disease
